# The Role of the *Prpk* Gene in the Body Development of *Apis cerana*

**DOI:** 10.3390/life15121906

**Published:** 2025-12-12

**Authors:** Xinru Zhang, Xinying Qu, Lingjun Xin, Hanbing Lu, Xiao Chen

**Affiliations:** 1 College of Bioscience and Resource Environment, Beijing University of Agriculture, Beijing 102206, China; 202330112016@bua.edu.cn (X.Z.); xinlingjun@bua.edu.cn (L.X.); 2 Institute of Apicultural Research, Chinese Academy of Agricultural Sciences, Beijing 100193, China; 82101235489@caas.cn (X.Q.); 821012530463@caas.cn (H.L.)

**Keywords:** *Apis cerana*, *Prpk*, bioinformatic analysis, sequence characterization, expression characteristics

## Abstract

*Apis cerana* is an important native honey bee species in China, and its body size is closely related to its production performance and environmental adaptability. *Prpk* (TP53-regulating kinase) has been confirmed to regulate cell growth and proliferation, thereby influencing body size development. However, its function in honey bees remains unclear. In this study, the protein structure and function of PRPK were analyzed, and the expression characteristics were examined at different developmental stages and tissues in Hainan Chinese honey bee and Aba Chinese honey bee. The molecular weight of this protein was 30.3 kDa, and the predicted isoelectric point was 9.13, and it had a conserved PKc_like superfamily domain. The sequence of PRPK was highly conserved from insects to mammals and fungi. The results of RT-qPCR showed that *Prpk* expression significantly increased during honey bee pupation, and its expression level was significantly higher in the larvae and early-stage pupae of the larger-bodied Aba Chinese honey bee. Additionally, *Prpk* exhibited the highest expression in the thoraces, suggesting its potential involvement in appendage development. This study indicated that *Prpk* may play a potential regulatory role in body size development in honey bees, providing a theoretical basis and candidate gene for elucidating the molecular mechanisms of body size formation and genetic improvement in honey bees.

## 1. Introduction

Honey bees are crucial pollinating and economic insects globally, making significant contributions in various aspects, such as product supply, ecological regulation, and cultural development [1,2,3,4]. The Chinese honey bee, *Apis cerana* Fabricius, is an indigenous bee resource in China. Compared to other introduced honey bee species, it exhibits a range of favorable biological traits, including an extended nectar-foraging period, efficiency in collecting scattered nectar sources, strong adaptability, varroa mite resistance, cold tolerance, and rapid comb construction [5,6,7,8]. These advantages make it widely utilized in apicultural production in China [9].

*A. cerana* in China is widely distributed across diverse geographical and climatic regions. Through long-term natural selection, these honey bees have become well-adapted to local environmental conditions, leading to the formation of honey bee populations with different body sizes [10]. For instance, the Aba Chinese honey bee is characterized by its relatively large body size and ability to adapt to high-altitude climates and nectar resource environments above 2000 m [11,12]. Meanwhile, on Hainan Island, located at the southern tropical margin, the unique geographical resources and climatic conditions have fostered the evolution of the Hainan Chinese honey bee population, which exhibits biological traits, such as a small body size, strong swarming tendency, and heat tolerance [13]. Body size traits are a major focus in honey bee breeding programs. As poikilothermic animals, the body size of honey bees is shaped by natural selection and phenotypic plasticity in response to environmental conditions [14,15]. Body size is closely correlated with environmental temperature [16]. Honey bees with a large body size have an advantage in surviving in cold climates and extend overwintering periods at high altitudes [17,18]. Individuals with large body sizes tend to exhibit high flight efficiency and possess a broad foraging range [19]. Moreover, they often demonstrate improved thermoregulatory capacity, enabling them to generate and conserve heat through morphological, physiological, or behavioral adaptations to maintain adequate body temperature under cold conditions [20]. There are diverse drivers and underlying mechanisms responsible for body size variation and adaptations, typically involving the conserved function of dietary and nutritional pathways, including insulin signaling and its downstream PI3K/TOR pathway [21,22].

TP53-regulating kinase (PRPK, also known as TP53RK), the human homolog of yeast piD261/Bud32, phosphorylates p53 at serine 15 and enhances its transcriptional activity. It was originally identified in activated lymphokine-activated killer (LAK) cells and has been proposed as a potential enhancer of adoptive immune cell function in cancer therapy [23]. In yeast, the homolog Bud32 is an atypical Ser/Thr kinase essential for normal cell growth and viability [24]. PRPK expression has been detected in highly proliferative human epithelial tumor cell lines, as well as in normal testis, with a modest increase observed during the G1 phase [25]. TP53RK is critical for the survival of colorectal cancer (CRC) cells, and its depletion inhibits CRC proliferation and induces a telomerase-associated DNA damage response [26]. Furthermore, studies in *Drosophila melanogaster* have shown that *Prpk* is ubiquitously expressed in imaginal discs, and its knockdown reduces cell size and impairs imaginal cell growth. In *Drosophila*, *Prpk* regulates cell growth and proliferation independently of p53 levels and requires the regulation of S6K activation through the PI3K/TOR pathway to maintain organ and body growth [27]. In mammals and *Drosophila*, the PI3K/mTOR pathway is a classic insulin-responsive pathway that regulates organ and cell size [28]. Significantly, mTOR was found to be the central player in both insulin-induced and nutrient-induced cell size changes [29,30]. One remarkable feature of the TOR pathway is its conservation as a major growth regulator in virtually all eukaryotes [31]. However, the role of *Prpk* within the PI3K/mTOR signaling pathway remains unexplored in honey bees.

In order to enrich basic information about the PRPK protein and elucidate its potential biological functions in *A. cerana*, we hypothesized that *Prpk* may be a candidate gene influencing honey bee body development. In this study, we first characterized the structural features and physicochemical properties of PRPK in *A. cerana*. Then, we investigated the cross-species conservation of this gene through sequence comparison and phylogenetic tree construction. Further, we examined the dynamic expression changes in *Prpk* at different developmental stages and different tissues in Hainan Chinese honey bee and Aba Chinese honey bee populations to lay the foundation for revealing its function in *A. cerana* body development. Our study provides foundational data for uncovering the mechanisms underlying honey bee body size regulation and offers a potential molecular target for honey bee breeding and genetic improvement.

## 2. Materials and Methods

### 2.1. Ethics Statement

The honey bee colonies used in this study were maintained by Chinese honey bee Conservation Farm. The ethics committee of the institute approved the experimental protocol (Approval No. MFSDWLLSC-2024-07; approval date: 6 August 2024).

### 2.2. Collection of Samples

Based on the physical characteristics of bees and the average annual temperature of their natural distribution areas, we collected samples of Aba Chinese honey bee (ABA) and Hainan Chinese honey bee (HAIN), which differ significantly in body size and living environment temperature. Samples of Aba Chinese honey bees were obtained from the Aba Honey Bee Conservation Farm in Maerkang City, Sichuan Province (102°7′12″ E, 31°54′4″ N), while samples of Hainan Chinese honey bees were obtained from the Hainan Honey Bee Conservation Farm in Wenchang City, Hainan Province (109°41′27″ E, 19°19′35″ N). Honey bee colonies were reared using standard beekeeping techniques.

**Figure 1:**
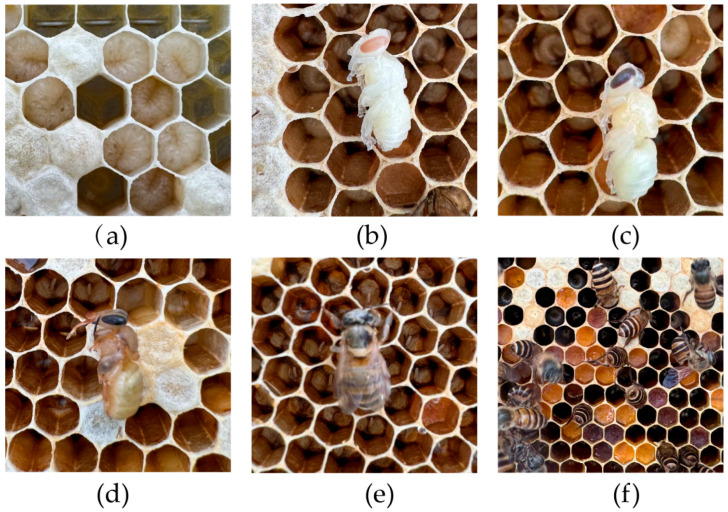
Samples of *A. cerana* at different developmental stages, using Hainan Chinese honey bee as an example. (**a**) Mature larvae, ML. (**b**) Early pupae, P1. (**c**) Mid-stage pupae, P2. (**d**) Late-stage pupae, P3. (**e**) New emerged bees, NEB. (**f**) Forager bees, FB.

To obtain samples at the same developmental stage, a colony was randomly selected, and the queen was caged to restrict it from laying eggs on a specific comb for 12 h. After that, the brood frame was marked and placed in a honey super for continued rearing, ensuring that the queen would not lay eggs on this frame again. The eggs hatched into larvae after 3 days, and they were then considered 1-day-old larvae. From this point, the larval age was calculated every 24 h until they emerged as adults. Samples of mature larvae (5-day-old), pupae (early, mid, and late stages), newly emerged bees, and forager bees were collected (*n* = 3, Figure 1). The collected larval samples were flash-frozen in liquid nitrogen and stored at −80 °C for the subsequent qRT-PCR. For the next tissue-specific expression detection, the collected adult bees were dissected to obtain three different tissues, including heads, thoraces, and legs. Dissections were performed on ice. Heads (thoraces and legs) from five bees were pooled as one biological sample (*n* = 3).

### 2.3. Bioinformatic Analysis

*Prpk* (LOC107999426) in *A. cerana* is located on chromosome 7 of AcerK_1.0 (GCF_029169275.1), ranging from 11,890,896 bp to 11,894,508 bp. The full length of this gene is 3613 bp. It contains 3 introns and encodes 249 amino acids. The physicochemical properties of the PRPK protein were analyzed using the ExPASy-ProtParam Tool (https://web.expasy.org/, accessed on 5 November 2025) to predict its amino acid length, isoelectric point, hydrophilicity and hydrophobicity, stability, etc. [32]. Its subcellular localization was predicted using WoLFPSORT (https://wolfpsort.hgc.jp/, accessed on 6 November 2025) [33]. The Conserved Domain Search function on the NCBI website (https://www.ncbi.nlm.nih.gov/cdd, accessed on 6 November 2025)was used to predict conserved regions of amino acids. The secondary and tertiary structures of PRPK were predicted using NPS@:SOPMA (https://npsa-prabi.ibcp.fr/, accessed on 7 November 2025) and SWISS-MODEL (https://swissmodel.expasy.org/, accessed on 7 November 2025).

### 2.4. Multiple Sequence Alignment and Phylogenetic Tree Analysis

To analyze the homology of PRPK among different species, we selected representatives from major biological groups, including *Drosophila melanogaster* (Dm), *Homo sapiens* (Hs), *Mus musculus* (Mm), *Arabidopsis thaliana* (At), and *Saccharomyces cerevisiae* (Sc), and downloaded the amino acid sequences of their PRPK homologous genes. The five PRPK amino acid sequences were subjected to multiple sequence alignment using DNAMAN 8.0. Next, we added the PRPK homologous gene amino acid sequences of *Apis mellifera*, *Apis laboriosa*, *Apis dorsata*, *Bombus terrestris*, and *Bombus pyrosoma* to construct a phylogenetic tree. A neighbor-joining phylogenetic tree was constructed using MEGA 7.0 with 1000 bootstrap replicates, while other parameters were set to the software defaults.

### 2.5. RNA Isolation and Real-Time Quantitative PCR

For the samples obtained as outlined in Section 2.1, total RNA was extracted and reverse-transcribed into cDNA, according to the supplier’s instructions, using the Trizol Up Plus RNA Kit (TransGen Biotech, Beijing, China, ER501-01-V2) and the PrimeScript™ RT Reagent Kit with gDNA Eraser (Perfect Real Time) (TaKaRa, Kusatsu, Shiga Prefecture, Japan, RR047A). Transcript-specific primer pairs (Table 1) were designed using NCBI Primer-BLAST (https://www.ncbi.nlm.nih.gov/tools/primer-blast/, accessed on 15 September 2025) and synthesized by Shanghai Sangon Biotech Co., Ltd. (Shanghai, China). The expression levels of *Prpk* in the samples were detected by qRT-PCR using the TB Green Premix Ex Taq™ II (Tli RNaseH Plus) kit (TaKaRa, RR820A) on the LineGene 9600 Plus Real-Time PCR System (Bioer Technology, Zhejiang Province, China), according to the manufacturer’s protocol^®^. The qRT-PCR reaction system had a total volume of 20 μL, consisting of 1 μL cDNA (200 ng, 1:5 dilution), 0.8 μL forward primer (10 μM), 0.8 μL reverse primer (10 μM), 10 μL TB Green Premix Ex Taq II, and 7.4 μL H_2_O. *β*-actin was used as the reference gene [32]. The cycling conditions were 95 °C for 30 s, followed by 45 cycles of 95 °C for 5 s and 60 °C for 30 s to acquire fluorescence signals. The results obtained from qRT-PCR were analyzed using the 2^−ΔΔct^ method based on Ct values. The data were analyzed using a one-way ANOVA in SPSS Statistics 27 software, with the TUKEY method employed for multiple comparisons. The final results are expressed as the mean ± standard deviation. Data visualization was conducted using Origin^®^ 2025b.

## 3. Results

### 3.1. The Physicochemical Properties and Structure of PRPK

The results of the assessment of the physicochemical properties of PRPK showed that the molecular weight of this protein is approximately 30.3 kDa (pI = 9.13), and its molecular formula is C_1370_H_2203_N_371_O_384_S_8_. There are 33 negatively charged residues (Asp + Glu), and there are 41 positively charged residues (Arg + Lys). There are a total of 4336 atoms, and the half-life of this protein is 30 h. The grand average of hydropathicity (GRAVY) is −0.262, and the aliphatic index is 110.77, indicating that PRPK is an alkaline water-soluble protein. Subcellular localization prediction suggested that it is a cytoplasmic protein. A conserved PKc_like superfamily domain, located at amino acid residues 32–253 (E-value = 6.91 × 10^−57^), was identified in the PRPK protein. This indicated that PRPK may be involved in regulating various cellular responses, including gene expression, protein secretion, cell proliferation, and inflammatory responses.

**Figure 2:**
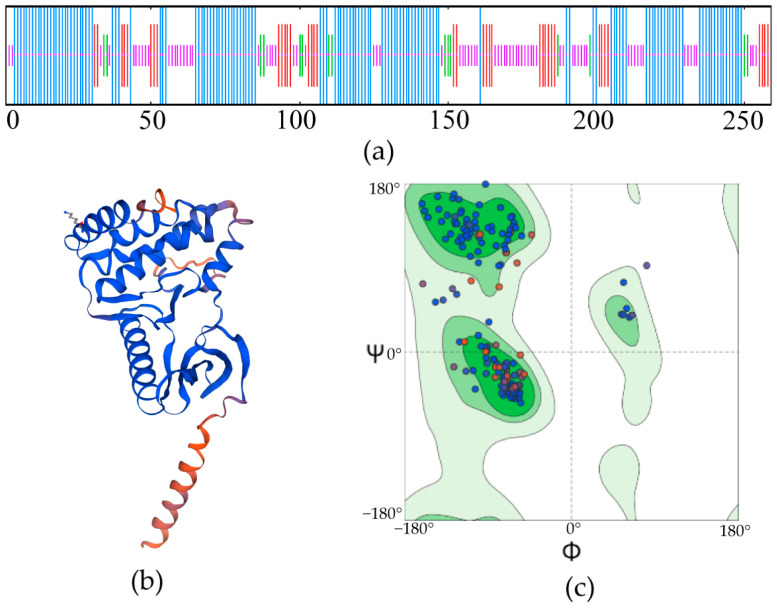
The secondary and tertiary structure prediction of the PRPK protein. (**a**) Secondary structure prediction. The α-helices (50.97%), irregular coils (37.45%), extend chains (11.58%), and β-turns (5.79%) are shown in blue, purple, red, and green, respectively. (**b**) Tertiary structure prediction. The model is presented in cartoon representation and colored by local prediction confidence (predicted local distance difference test, pLDDT score): dark blue (high confidence, >90), light blue (good confidence, 70–90), yellow (medium confidence, 50–70), and orange-red (low confidence, <50). (**c**) The ramachandran map analysis of the tertiary structural model. The most favored, additionally allowed, generously allowed, and disallowed regions are shaded in dark green, green, light green, and white, respectively. The blue dots represent standard amino acid residues (excluding glycine and proline residues). The red dots represent glycine or proline residues. The purple dots represent outlier residues that may require further validation. Figure 2a: Reprinted with permission from Ref. [35], 2016, PBIL-IBCP-Lyon. Figure 2b: Reprinted with permission from Ref. [36], 2024, The Computational Structural Biology Group at the SIB Swiss Institute of Bioinformatics at the Biozentrum, University of Basel. The Raman diagram analysis of the prediction model was conducted using The Structure Analysis and Verification Server (https://saves.mbi.ucla.edu/, accessed on 7 November 2025). Figure 2c: Reprinted with permission from Ref. [37], 2004, Prof. Gert Vriend.

The prediction of the secondary structure of PRPK revealed that α-helices accounted for 50.97% (132 amino acids), irregular coils accounted for 37.45% (97 amino acids), and extended chains accounted for 11.58% (30 amino acids) (Figure 2a). The tertiary structure of PRPK showed that the GMQE was 0.65, the QMEAN was 0.67, and the similarity of the sequence to the human PRPK complex 7szd. 1. A was 39.91%. There were many α-helices in the tertiary structure of proteins, which were rich in structure (Figure 2b). Raman diagram analysis indicated that the amino acid residues located in the core region (typically the central dark green area) accounted for 92.2% of the entire protein. It could be considered that the conformation of this model conforms to the rules of stereochemistry (>90%).

### 3.2. Amino Acid Sequence Alignment of PRPK

**Figure 3:**
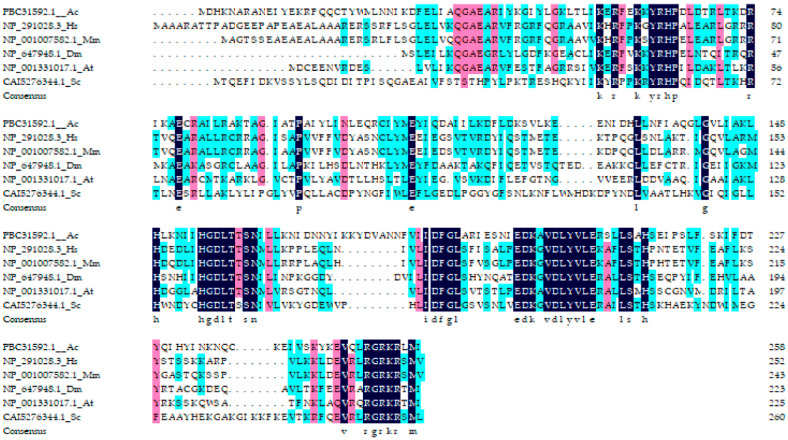
Multiple alignment of PRPK. Different colors represent the conservation rate of amino acid sequences among six species. Black represents the amino acid sequences conservation is 100% among these species. Pink represents the amino acid sequences conservation among these species is at least 80%. Blue represents the amino acid sequences conservation among these species is less than 80%.

The results of the amino acid sequence alignment of PRPK showed that despite spanning multiple biological groups, the overall homology among these six species was relatively high, reaching 50.84% (Figure 3). The amino acid sequence consistency of PRPK with the other five species from high to low was *Drosophila melanogaster* > *Arabidopsis thaliana* > *Homo sapiens* > *Mus musculus* > *Saccharomyces cerevisiae*. Among them, the amino acid sequence consistency between *Drosophila melanogaster* and *A. cerana* was the highest, at 43.59%, while the PRPK amino acid sequence consistency with yeast was the lowest, at 32.28%.

### 3.3. Phylogenetic Analysis

**Figure 4:**
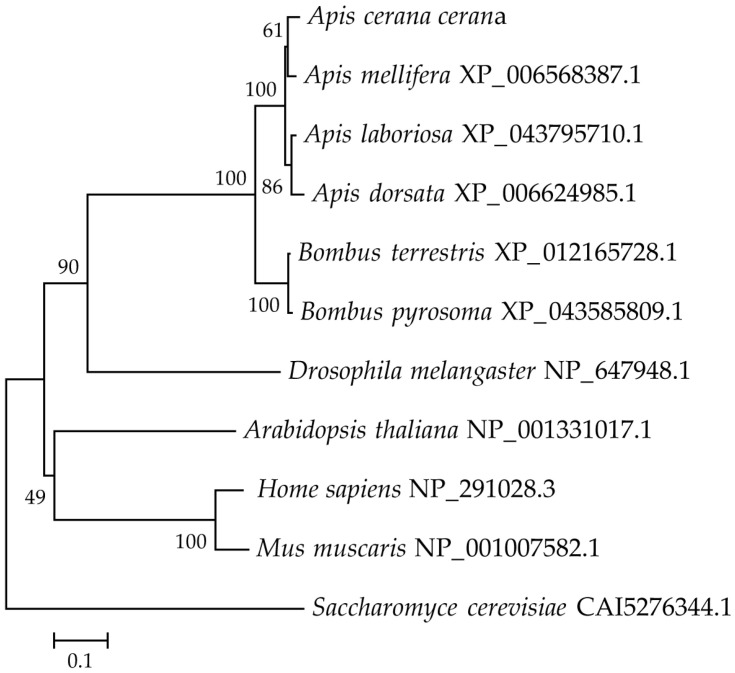
Phylogenetic tree of PRPK between *A. cerana* and ten other species constructed based on amino acid sequence by neighbor-joining method (1000 replicates).

The results of the phylogenetic tree analysis showed that PRPK is mainly divided into five major branches, including fungi, mammals, plants, and Diptera and Hymenoptera insects, during the evolution of different species. Except for Hymenoptera, humans and mice clustered together into one branch, and the others formed separate branches. However, the amino acid sequence of *A. cerana* was the closest to those of *Apis mellifera*, *Apis laboriosa*, and *Apis dorsata*, which belong to the same genus of honey bees, and the furthest evolutionary distance from yeast (Figure 4).

### 3.4. The Expression Characteristics of Prpk in Aba Chinese Honey Bee and Hainan Chinese Honey Bee

#### 3.4.1. Dynamic Changes in Prpk Gene Expression

**Figure 5:**
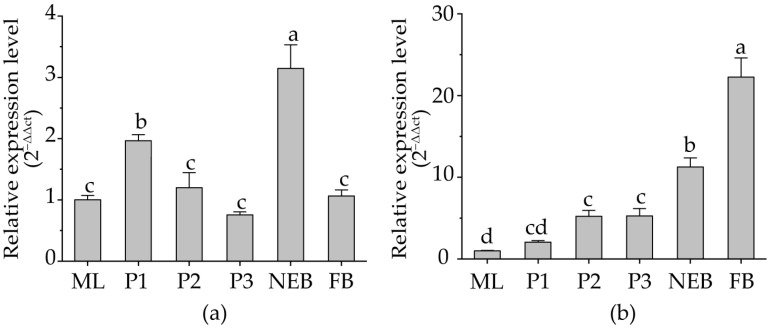
Dynamic expression of *Prpk* in Aba Chinese honey bee and Hainan Chinese honey bee at different developmental stages. Values are presented as mean ± standard deviation. (**a**) ABA, Aba Chinese honey bee. (**b**) HAIN, Hainan Chinese honey bee. ML, mature larvae. P1, early-stage pupae. P2, mid-stage pupae. P3, late-stage pupae. NEB, new emerged bees. FB, forager bees. Note: Values followed by different letters (a, b, c, d) are significantly different at *p* < 0.05.

The results of qRT-PCR showed that *Prpk* was expressed continuously in all developmental stages of honey bees, while the expression trend was slightly different in ABA and HAIN (Figure 5). In ABA, the expression of *Prpk* showed two peaks at the mid-pupal stage and in newly emerged bees, with the highest expression level in newly emerged bees. In HAIN, the expression level of *Prpk* gradually increased with the development process, especially after a period of field collection, and it was significantly higher than that in mature larvae (*p* < 0.05). Interestingly, the expression of *Prpk* in both ABA and HAIN increased during the larva pupation stage (*p* < 0.05), which indicated that *Prpk* may play a role in larval development. This may be related to the differentiation of bee segments during the transition period from larva to pupa. Additionally, it is worth noting that the expression of *Prpk* in foraging bees decreased significantly compared with the honey bees newly emerged in ABA, while it was significantly increased in foraging bees in HAIN. This indicated that *Prpk* may exert different physiological activity regulatory functions in different species.

#### 3.4.2. Tissue Expression Specificity of Prpk

**Figure 6:**
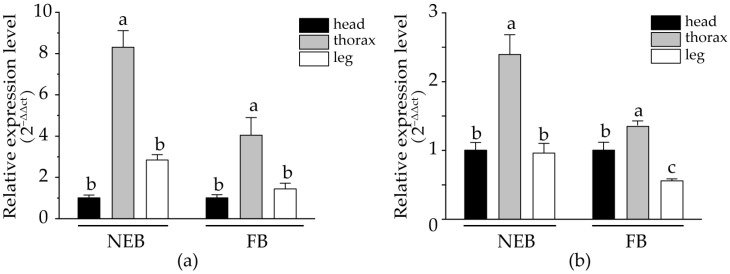
Tissue-specific expression of *Prpk* in Aba Chinese honey bee and Hainan Chinese honey bee. Values are presented as mean ± standard deviation. (**a**) Aba Chinese honey bee. (**b**) Hainan Chinese honey bee. NEB, new emerged bees. FB, forager bees. Note: Values followed by different letters (a, b, c) are significantly different at *p* < 0.05.

The qRT-PCR results showed that *Prpk* was expressed in the heads, thoraces, and legs of adult bees (Figure 6). Regardless of the breeds and periods, the expression of *Prpk* in the thoraces was significantly higher than that in the heads and legs (*p* < 0.05). This might be because bees have many appendages on their thorax, and the cell proliferation and differentiation involved in the appendage development process require the participation and function of *Prpk*.

#### 3.4.3. Comparison of Prpk Expression Between Aba Chinese Honey Bee and Hainan Chinese Honey Bee

**Figure 7:**
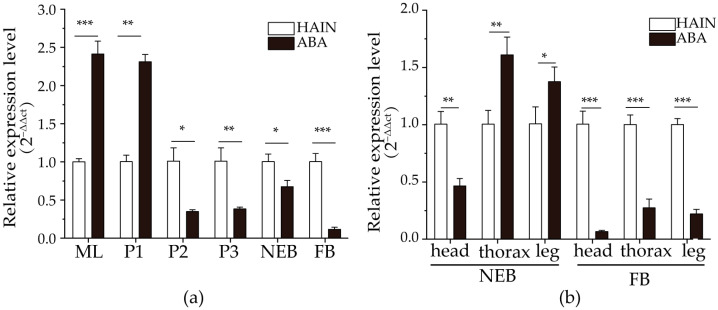
Comparison of *Prpk* gene expression levels between Aba Chinese honey bee and Hainan Chinese honey bee. (**a**) Comparison of *Prpk* expression in different developmental stages of Aba Chinese honey bee and Hainan Chinese honey bee. (**b**) Comparison of *Prpk* expression in different tissues of Aba Chinese honey bee and Hainan Chinese honey bee. HAIN, Hainan Chinese honey bee. ABA, Aba Chinese honey bee. ML, mature larvae. P1, early-stage pupae. P2, mid-stage pupae. P3, late-stage pupae. NEB, new emerged bees. FB, forager bees. Note: *, *p* < 0.05; **, *p* < 0.01; ***, *p* < 0.001.

At the larval and early pupal stage, the expression of *Prpk* in the large-bodied Aba Chinese honey bee was significantly higher than that in HAIN (*p* < 0.05) (Figure 7). However, from the mid-pupal stage to the period when bees go out to forage, the gene expression in HAIN was higher than that in ABA. The results indicated that during the critical period of honey bee morphological differentiation, *Prpk* may be a component of the mechanism that promotes large body growth, allowing cell mass to accumulate in larval tissues and promoting cell growth and proliferation in the limb imaginal discs.

## 4. Discussion

As a pivotal pollinator and a model organism, the honey bee plays a critical role in ensuring food security and maintaining ecological balance. Healthy and strong colonies exhibit enhanced reproductive capacity and pollination efficiency, thereby improving their productivity in terms of apicultural goods [35]. Worker bees are the most numerous individuals in a honey bee colony, and they are responsible for the vast majority of tasks within the colony [9]. Therefore, the health, lifespan, and foraging ability of worker bees are crucial to the development of the entire colony. Previous studies showed that large workers generally possess great productive potential, manifesting as long lifespans, strong foraging and flight abilities, and strong robust immune function [36,37]. Hence, identifying genes associated with body development and body size has become a major objective in honey bee breeding programs.

Many molecular markers and genes associated with body size have been identified in livestock and poultry. For instance, Wang et al. discovered that *Aff4* is a key gene driving latitudinal adaptation and body length variation in pigs [38]. Insulin-like growth factor 2 mRNA-binding protein 1 (*Igf2bp1*) has been identified as a major gene responsible for the larger body size in Beijing ducks. During the breeding process of the duck, a natural mutation occurred in a distal enhancer of this gene, causing it to continue being expressed after hatching, and the improved feed efficiency has led to an increase in body size [39]. The forkhead box O (*Foxo*) affects the growth rate and developmental stages of silkworms by regulating JH degradation and hormonal homeostasis, ultimately leading to a smaller body size and precocious sexual maturity [40]. Tp53-regulating protein kinase (PRPK) has been identified as a protein involved in proliferation. *Prpk* can regulate cell growth and proliferation by modulating S6K activation in a TOR-dependent manner, thereby maintaining organ growth in fruit flies to achieve the final body size, but its role in honey bees has rarely been reported before [27].

Our study identified the genetic characteristics of *Prpk* in *A. cerana*. The PRPK protein contained a high proportion of α-helices, making the overall protein structure relatively stable and less prone to unwinding [41]. Multiple sequence alignment revealed that PRPK shares up to 50% similarity among different species, indicating that this protein is a highly conserved core protein, and its biological function is essential for normal species survival. mTOR nucleates two functionally distinct complexes, mTOR complex 1 (mTORC1) and mTORC2 [27,42]. The conserved domain, the protein kinase C (PKC) family, belongs to the AGC kinase family, which has been well-characterized as an mTORC2 substrate. PRPK has a PKc_like superfamily domain, which implies that PRPK may exhibit characteristics of the PKC family and play a role in cell proliferation, differentiation, and other processes through the TOR signaling pathway [29,31,43]. The RT-qPCR results indicated that during the metamorphic transition from larvae to pupae, the expression of *Prpk* significantly increased. During this period, the external segments of the honey bee begin to form, while specific tissues in the larvae undergo programmed cell death and remodeling, generating adult tissues such as the fat body and midgut from the imaginal discs [44,45]. These specific tissues will develop into pupal organs through processes like autophagy, apoptosis, rearrangement, and differentiation [46]. In addition, we observed that the expression level of *Prpk* varied among the heads, thoraces, and legs of honey bee, with the highest expression in thoracic tissues. It is speculated that *Prpk* may be involved in regulating cell growth and differentiation in various appendages and exoskeleton structures, such as the legs and wings that develop from the imaginal discs in the thorax [47,48]. A previous related study in *Drosophila* showed that the generalized knockdown of *Prpk* produced a clear reduction in larval size [27]. By quantifying the number of hairs (each cell produces one) present in a defined area in adult wings and the 5-Bromo-2′-deoxyuridine (BrdU) incorporation rate, it was found that knocking down *Prpk* reduced cell body size and impaired proliferation, both of which contribute to the final decrease in tissue size [27]. Similarly, comparisons between the Aba Chinese honey bee and Hainan Chinese honey bee in terms of larval growth showed that the expression of *Prpk* was significantly higher in the large Aba Chinese honey bee than that in Hainan Chinese honey bee, suggesting that it may be related to honey bees’ somatic cell size, development, and response to environmental stimuli. Furthermore, the best described mTORC1 substrates are S6K and 4E-BP [43]. *Prpk* knockdown in Drosophila inhibited S6K and 4E-BP phosphorylation. Co-expression with an activated form of S6K completely abolished cell growth deficiency, suggesting that *Prpk* could be upstream of S6K activation [49]. Our gene expression experiments conducted in the differently sized Aba Chinese honey bee and Hainan Chinese honey bee suggest that *Prpk* may be an important regulator involved in the mTOR signaling pathway, thereby influencing honey bee body development. Further studies are needed to focus on functional perturbation experiments to explore its downstream pathways or interacting proteins to clarify the molecular mechanism behind this regulation.

## 5. Conclusions

In summary, this study analyzed *Prpk*’s protein structure and properties using bioinformatic methods, finding that it has a PKc_like superfamily domain and is conserved across multiple species. Compared to mature larvae, the expression level of *Prpk* in Aba Chinese honey bee showed two peaks at the pupation and emergence period of honey bees. The expression of *Prpk* in Hainan Chinese honey bee gradually increased with the development process. The tissue-specific results showed that the expression of *Prpk* is the highest in the thorax. The experimental results indicated that it may play an important role in larval metamorphosis into pupae, as well as in the growth of the thoracic limbs and exoskeleton, and it may influence the final body size of bees by regulating cell size or proliferation. This study not only found that *Prpk* is involved in body size development in honey bees but also provided a new gene target for the marker-assisted breeding of honey bees.

## Figures and Tables

**Table 1:** Primer sequences used in this study.

Primer Name	Primer Sequences	Ref.
Prpk-F	5′-TTTCTCAGCTTTAATACGATGTTGA-3′	Designed by this study
Prpk-R	5′-TTATATATACGTGCTTCAGCGCC-3′	
β-actin-F	5′-CTGCTGCATCATCCTCAAGC-3′	[34]
β-actin-F	5′-GAAAAGAGCCTCGGGACAAC-3′	

## Data Availability

No new data were created or analyzed in this study. Data sharing is not applicable to this article.
